# Postoperative intensity‐modulated radiation therapy reduces local recurrence and improves overall survival in III‐N2 non‐small‐cell lung cancer: A single‐center, retrospective study

**DOI:** 10.1002/cam4.2937

**Published:** 2020-02-26

**Authors:** Wei Wei, Jiao Zhou, Qun Zhang, De‐Hua Liao, Qiao‐Dan Liu, Bei‐Long Zhong, Zi‐Bin Liang, Yong‐Chang Zhang, Rong Jiang, Gui‐Yun Liu, Chen‐Yang Xu, Huai‐ Li Zhou, Su‐Yu Zhu, Nong Yang, Wen Jiang, Zhi‐Gang Liu

**Affiliations:** ^1^ The Cancer Center of the Fifth Affiliated Hospital of Sun Yat‐Sen University Zhuhai China; ^2^ Guangdong Provincial Key Laboratory of Biomedical Imaging The Fifth Affiliated Hospital Sun Yat‐sen University Zhuhai China; ^3^ Department of Radiation Oncology Hunan Cancer Hospital The Affiliated Cancer Hospital of Xiangya School of Medicine Central South University Changsha China; ^4^ Department of Clinical Medicine University of South China Hengyang China; ^5^ Department of Radiotherapy First Affiliated Hospital of Sun Yat‐sen University Guangzhou China; ^6^ Department of Pharmacy Hunan Cancer Hospital The Affiliated Cancer Hospital of Xiangya School of Medicine Central South University Changsha China; ^7^ Department of Thoracic Surgery The Fifth Affiliated Hospital Sun Yat‐sen University Zhuhai China; ^8^ Department of Medical Oncology, Lung Cancer and Gastrointestinal Unit Hunan Cancer Hospital/The Affiliated Cancer Hospital of Xiangya School of Medicine Central South University Changsha China; ^9^ Department of Radiation Oncology The University of Texas Southwestern Medical Center Dallas USA

**Keywords:** local recurrence, locally advance, non‐small‐cell Lung Cancer, postoperative radiotherapy

## Abstract

**Purpose:**

To determine the postoperative effects of radiotherapy (PORT) on the local recurrence‐free survival (LRFS) and overall survival (OS) of stage III‐N2 non‐small‐cell lung cancer (NSCLC).

**Materials and Methods:**

183 patients with resected stage III‐pN2 NSCLC from Hunan Cancer Hospital between 2013 and 2016 were divided into two groups for postoperative chemotherapy (POCT) (n = 105) or combination chemotherapy and radiotherapy (POCRT) (n = 78). The LRFS and OS were compared and the factors affecting local recurrence were illustrated in these two groups. The sites of failure based on the lobe of the primary tumor in two groups were described.

**Results:**

PORT leads to a strikingly lower risk for local recurrence and brought superior OS benefit. For different pN2 Subclassification, Patients with multiple‐station pN2 ± pN1 disease had the worst LRFS (11 months) and single‐station pN2 + multiple station pN1 disease had a relatively short LRFS (24 months) in group POCT. Short LRFS is correlated with multiple‐station pN2, older age (Y > 55), patients with a high positive LN ratio > 1/3 and a poor tumor histological differentiation degree. In group POCT, the most frequent failure site occurs at the ipsilateral hilum (21.0%), the bronchial stump (20.0%), followed by LNs4R (19.0%), LNs4L (18.1%), LNs7 (15.2%), most of left‐sided tumors more frequently involved the contralateral mediastinum, whereas the ipsilateral recurrences dominated for right‐sided tumors, especially for LNs4R. In group POCRT, the highest failure site was the bronchial stump (11.5%), followed by LNs4L (8.97%), LNs1 (7.69%), the ipsilateral hilum (6.41%) and LNs4R (6.41%).

**Conclusion:**

PORT remarkably reduced local recurrence and improved OS in stage III‐pN2 NSCLC, especially in the multiple‐station pN2 group.

## Graphical abstract

This retrospective analysis included 183 patients with resected stage III‐pN2 NSCLC in Hunan Cancer Hospital between 2013 and 2016. They were divided into two groups for postoperative chemotherapy (POCT) (n = 105) or combination chemotherapy and radiotherapy (POCRT) (n = 78). The local recurrence‐free survival (LRFS) and overall survival (OS) of two groups were compared and illustrated the factors affecting local recurrence in group POCT. The results showed that PORT significantly reduced locoregional relapse (LR) and improved OS in stage III non‐small cell lung cancer.

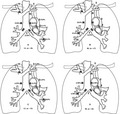

## 1 INTRODUCTION

Lung cancer has the highest incidence in solid tumors and has been considered as the leading cause of cancer‐related mortality worldwide.^1^ NSCLC represented approximately 80% of all lung cancers.^2^ Surgery is the mainstay of therapy for the resectable stage III‐pN2 NSCLC, but local relapse and distant metastasis (DMs) would still occur after surgery frequently, disease recurrence after surgical resection reduces the patients’ life expectancy sharply.^3^ Researches show that Postoperative chemotherapy can reduce distant metastasis for patients with postoperative pathologically involved positive nodes.^4^ However, the risk of locoregional recurrence reached as evidently as 20%‐40% even after complete resection, adjuvant and postoperative chemotherapy.^2^

PORT is an appealing means of decreasing locoregional recurrence, however, whether it can convert into survival benefit remains controversial.^5^ Studies have been performed to evaluate the role of PORT on OS of patients with resectable stage III‐pN2 NSCLC. A meta‐analysis illustrated that outmoded radiotherapy techniques, doses, and large radiotherapy fields from PORT in 1998 lead to cardiac and pulmonary toxicity and caused a 7% absolute increase in mortality.^6^ Further study, however, has bolstered the use of PORT with modern conformal radiation therapy (CRT) techniques was associated with improved survival for patients with N2 disease,^5^, ^7^ based on the outcomes of META studies, PORT offers advantages in local recurrence rate, but continue to show a detrimental effect on overall survival.^8^ A randomized clinical trial^9^ evaluating the efficacy of PORT is ongoing (LUNG ART) and is expected to provide explicit guidance for pN2 NSCLC.

The objective of PORT is to reduce local recurrence (LR) and to translate into a survival benefit. Accordingly, the issue of postoperative local recurrence and who may benefit from PORT may soon carry increasingly important.^10^ Defined high‐risk of local recurrence group can improve the pertinence of PORT, many researchers have indicated that in patients with pN2 NSCLC, the number of pN2 lymph nodes, Nodal Stage, Pathologic T stage may be tightly associated with survival outcomes.^10^, ^11^ Presently, there are no prospective data that describe the risk for LR of pN2 NSCLC after surgical resection, and risk factors for LR are not well defined.^12^

If PORT is to be successful, optimal target volumes ought to be defined. However, specialists have come to no consensus regarding which lymph node regions of completely resected NSCLC patients to include in the CTV for three‐dimensional (3D)‐CRT treatment.^13^ PORT CTV for different sided lung cancers needs to be based on comprehensive surgical, nodal involvement distribution, and mediastinal lymphatic drainage radiographic evidence.^14^, ^15^ In 2010, the article summarized in detail which lymph node regions needed to be included in the delineation of CTV for different positive lymph nodes after surgery.^16^ According to the definition of CTV in different cancer centers, it basically includes stump, ipsilateral hilum, positive lymph nodes, and subcarinal lymph nodes. Currently, an “electively limited RT field” is accepted by most of the clinicians.

Therefore, this study aims to further verify and analyze the efficacy of PORT, find patients with high postoperative local recurrence risk and explore the locoregional relapse patterns to provide patients with R0 resected stage III‐pN2 NSCLC a reference for PORT CTV delineation.

## 2 MATERIALS AND METHODS

### 2.1 Patient characteristics

The retrospective data collection of this study was approved by the institutional review board in Hunan cancer hospital. Included in the analysis were 183 adult patients (age > 20 years) with pathologically confirmed stage III‐pN2 NSCLC who underwent complete resection between 2013 and 2016. According to whether they received PORT or not, all the 183 patients were divided into two groups for POCT (n = 105) or POCRT (n = 78). The patients received a score of ECOG ≤ 2, brain MRI, ECT and abdominal ultrasound showed no distant metastasis before surgery. Inclusion of the study complied with the following predetermined standard: complete resection of NSCLC with pathological confirmed stage III (N2); margin negative resection of all gross disease; Available imaging data at the first time of recurrence or metastasis and patients who received adjuvant (chemotherapy and/or RT). Patients who received neoadjuvant therapy (chemotherapy and/or RT), adjuvant targeted therapy only and patients with simultaneous or sequential second primary tumor were excluded.

### 2.2 Treatment (surgery, postoperative chemotherapy, and radiotherapy)

Surgical procedures include radical lung resection, including lobectomy or pneumonectomy and systemic mediastinal lymph node dissection. Platinum‐based adjuvant chemotherapy was performed alone or with radiotherapy with a median of four cycles (range, one to six) begin with 3‐4 weeks after surgery. In patients receiving PORT, intensity‐modulated radiotherapy (IMRT) was used. The CTV for treatment generally encompassed the bronchial stump, the initially involved mediastinal LNs, the ipsilateral hilum and the subcarinal region (station 7) and the supraclavicular fossae were not included in the routine. Dose‐volume constraints for OARs (organ at risk) was basically accorded with the standard, total lung V20 < 20%, spinal cord Dmax < 45Gy, cardiac V30 < 40%, V40 < 30%, esophageal V50 < 50%. Radiotherapy was delivered with linear accelerators, using total dose ranging from 48 Gy to 54 Gy, and a median dose of 50 Gy of 6 to 8 MV X‐ rays at 1.8‐2.0 Gy per fraction, 5 days per week.

### 2.3 Follow‐up

Patients were generally followed every 3 months after surgery for the first 2 years and every 6‐12 months thereafter. Standard follow‐up evaluation included a physical examination, biochemical tests, chest computed tomography (CT) scans, brain CT scan and ultrasonograms or CT scans of the abdomen. Clinical assessments, imaging studies, and pathology reports were used for the determination of treatment failure. Follow‐up information was obtained by reviewing electronic medical records in the clinic and by conducting telephone surveys. The end date of the follow‐up was December 31, 2018.

### 2.4 The evaluation index

Our study's primary objective was to explore the Patterns of the first local recurrence (LR), the local recurrence‐free survival (LRFS) which was defined as the period of time from surgery to local recurrence and overall survival (OS), which was defined as the time period from surgery to death or the date of last visit. LR was identified as a relapse including the bronchial stump, hilar, mediastinal, subclavicular or supraclavicular LNs; all other sites failure were considered as distant metastases (DMs). Nodal failures are considered to meet the following three points: (a) when a new or enlarging lymph node measuring larger than 1 cm in the short axis on CT, (b) the enlarged lymph nodes gradually increase or shrink after anti‐tumor treatment, (c) hypermetabolic on positron‐emission tomography (PET) or confirmed by pathology (regardless of size). We evaluated the Sites of tumor recurrence within the mediastinum or hilum, according to the 2009 IASLC lymph node map.^17^ Staging occurred according to the TNM classification in the Union for International Cancer Control (UICC) 8th ed.^18^

### 2.5 Statistical analysis

The Pearson *χ* ^2^ test was used to determine unadjusted associations between group POCRT and POCT. The crude analysis of OS or LRFS of two groups was estimated by using the Kaplan‐Meier method. The univariate association of each covariate with LRFS was assessed using log‐rank tests. Multivariable Cox proportional hazards models were used to calculating adjusted hazard ratios (HRs) and their 95% CIs relating to the variables as described. Results were considered to be statistically significant when *P < *.05.

## 3 RESULTS

### 3.1 Clinical characteristics

**Figure 1:**
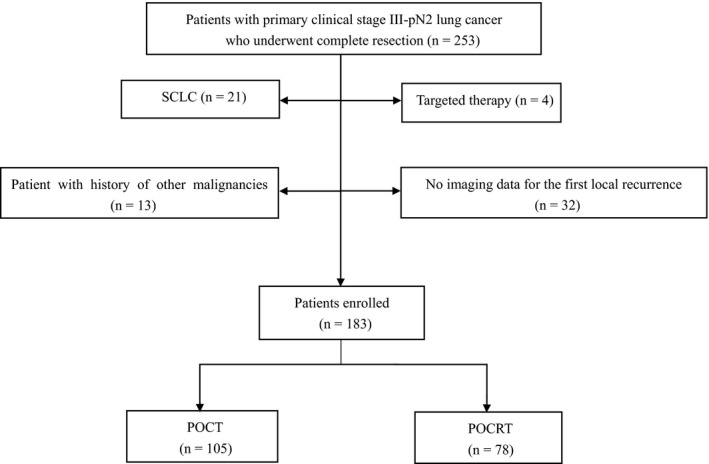
Selection of patients

According to the selected standard, the analysis comprises a total of 183 patients. 78 patients received POCRT (group POCRT) and 105 patients received POCT (group POCT). Figure 1 describes the information. The median patient age at diagnosis for the patients within the cohort was 55 years (range from 39 to 75), the median follow‐up time is 38 months (range from 4 to 67). We analyzed the updated data on two groups for survival, local recurrence, and the last follow‐up or date of death, as well as detail pieces of information of the treatment, tumor stage, status of LNs, number of LNs, gender, age, and smoking status. Except for the number of Local recurrences (*P < *.001) and the age (*P* = .031), no significant differences in clinical characteristics were found between the two groups. Table 1 describes the specific information.

**Table 1:** 183 patients clinicopathological characteristics statistics according to receipt of PORT [n (%)]

Characteristic	Total (n = 183)	PORT		*χ* ^2^	*P*
		No (n = 105)	Yes (n = 78)		
Gender				0.124	.725
Male	117 (63.9)	66 (62.9)	51 (65.4)		
Female	66 (36.1)	39 (37.1)	27 (34.6)		
Age				4.650	.031
Y > 55	92 (50.3)	60 (57.1)	32 (42)		
Y ≤ 55 y	91 (49.7)	45 (42.9)	46 (59)		
Histology				0.161	.923
Squamous carcinoma	54 (29.5)	30 (28.6)	24 (22.9)		
Adenocarcinoma	111 (60.7)	65 (61.9)	46 (43.8)		
Others	18 (9.8)	10 (9.5)	8 (7.6)		
Smoke				0.099	.752
Yes	101 (55.2)	59 (56.2)	42 (53.8)		
No	82 (44.8)	46 (43.8)	36 (46.8）		
Laterality				0.443	.506
Left	70 (38.3)	38 (36.2)	32 (41.0)		
Right	113 (61.7)	67 (63.8)	46 (59.0)		
Operation					
Wedge resection	12 (6.60)	8 (7.62)	4 (5.10)	0.683	.706
Lobectomy	160 (87.4)	90 (85.7)	70 (89.7)		
Pneumonectomy	11 (6.0)	7 (6.67)	4 (5.10)		
Adjuvant chemotherapy				3.234	.072
Cycle < 4	45 (24.6)	31 (29.5)	14 (17.9)		
Cycle ≥ 4	138 (75.4)	74 (70.5)	64 (82.1)		
Differentiation degree				0.257	.880
Well	19 (10.4)	10 (9.5)	9 (11.5)		
Moderately	104 (56.8)	61 (58.1)	43 (55.1)		
Poorly	60 (32.8)	34 (32.4)	26 (33.3)		
T stage				3.565	.168
T1	81 (44.3)	46 (43.8)	35 (44.9)		
T2	74 (40.4)	47 (44.8)	27 (34.6)		
T3	28 (15.3)	12 (11.4)	16 (20.5)		
T size				0.099	.752
T ≤ 3 cm	82 (44.8)	46 (43.8)	36 (46.2)		
T> 3 cm	101 (55.2)	59 (56.2)	42 (53.8)		
N2				0.400	.527
Multiple station N2	103 (56.3)	57 (54.3)	46 (59)		
Single station N2	80 (43.7)	48 (45.7)	32 (41)		
Locoregional recurrence				12.860	<.001
Yes	67 (36.6)	50 (47.6)	17 (21.8)		
No	116 (63.4)	55 (52.4)	61 (78.2)		
Distant metastasis				1.382	.240
Yes	103 (56.3)	63 (60.0)	40 (51.3)		
No	80 (43.7)	42 (40.0)	38 (48.7)		
Die				3.377	.066
Yes	80 (43.7)	52 (49.5)	28 (35.9)		
No	103 (56.3)	53 (50.5)	50 (64.1)		

Abbreviations: PORT, Postoperative Radiotherapy.

### 3.2 LRFS and OS

**Figure 2:**
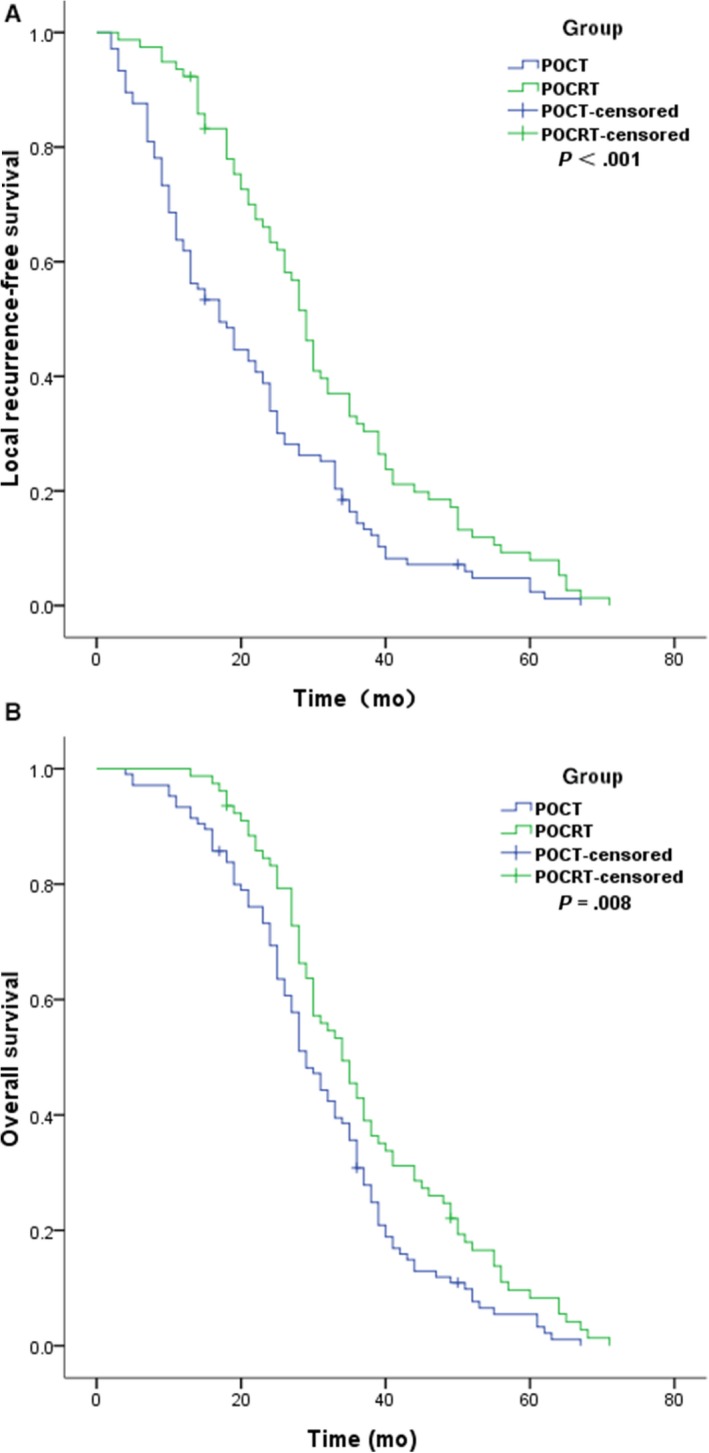
Comparison of local recurrence‐free survival (A) and overall survival (B) between the PORT and the POCRT group

LRFS and OS were compared between the POCRT and the POCT groups. Median and 2‐year LRFS values were significantly higher in patients of group POCRT versus group POCT (median LRFS, 29 versus 17 months; 2‐year LRFS, 62.1% versus 35.1%; *P *< .001) (Figure 2A). Median and 2‐year OS also values significantly improvement in patients of group POCRT versus group POCT (median OS, 34 versus 29 months; 2‐year OS, 78.3% versus 62.1%; *P* = .008) (Figure 2B). During the course of the disease, 50 patients (47.6%) (50/105) in group POCT and 17 patients (21.8%) (17/78) in group POCRT represented a first site or cumulative LR. The ratio of LRs emerged as the first site and concomitant with distant relapse was 36.2% (38/105) in group POCT and 16.7% (13/78）in group POCRT, most of the patients with an LR also had DMs (distant metastasis) at the time of diagnosis of an LR. The ratio in group POCT and POCRT was, respectively, (76.0%) (38/50) vs (76.5%) (13/17). Most LR was proved by CT in all patients and by pathological examination or FDG‐PET/CT in a proportion of patients.

### 3.3 pN2 subclassification and LRFS

**Figure 3:**
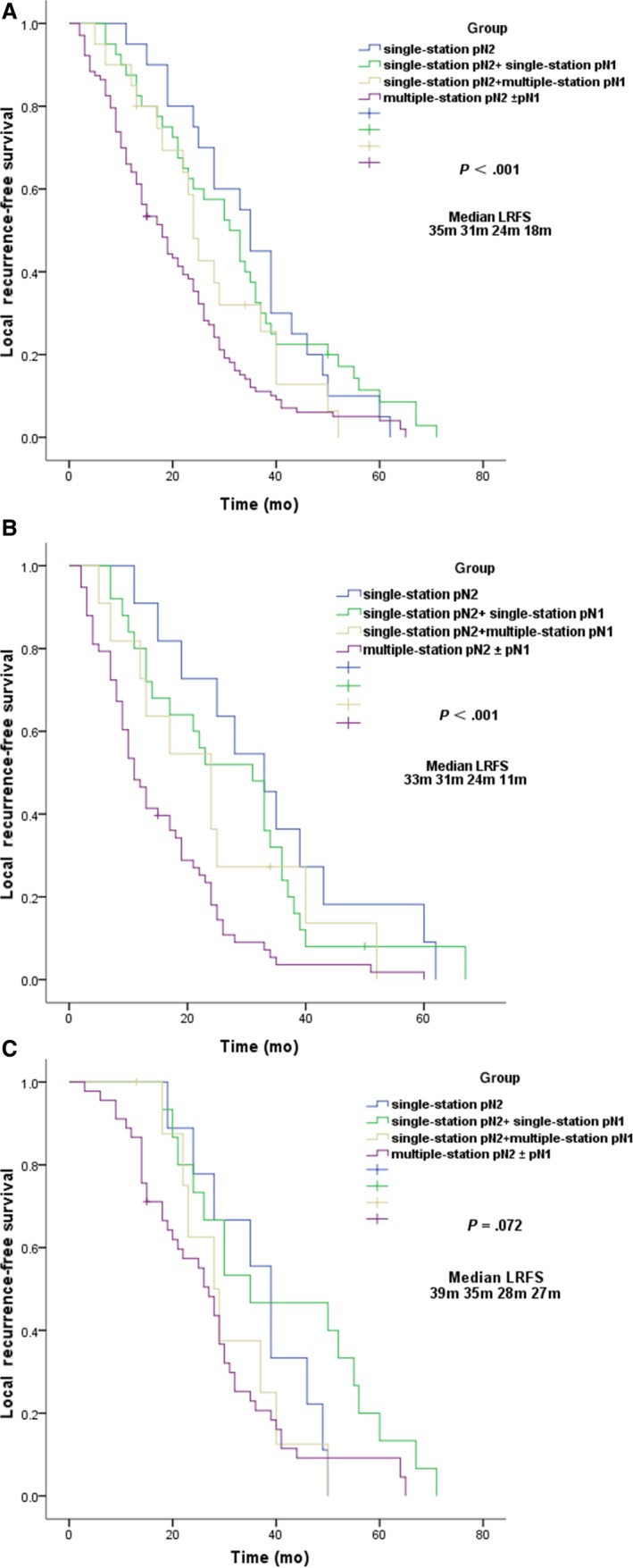
Comparison of local recurrence‐free survival among the group single‐station pN2, single‐station pN2 + single‐station pN1, single‐station pN2 + multiple‐station pN1 and multiple‐station pN2 ± pN1 of 183 patients (A); Comparison of local recurrence‐free survival among the group single‐station pN2, single‐station pN2 + single‐station pN1, single‐station pN2 + multiple‐station pN1 and multiple‐station pN2 ± pN1 in group POCT (B); Comparison of local recurrence‐free survival among the group single‐station pN2, single‐station pN2 + single‐station pN1, single‐station pN2 + multiple‐station pN1 and multiple‐station pN2 ± pN1 in group POCRT (C)

The local recurrence prognostic relevance of the pN2 subclassifications of 183 patients was evaluated. They were divided into a single‐station pN2, a single‐station pN2 + single‐station pN1 group, a single‐station pN2 + multiple‐station pN1 group, and a multiple‐station pN2 ± pN1 group. The subclassification of patients with metastatic pN2 lymph nodes was significantly associated with LRFS, the Median LRFS was, respectively, 35, 31, 24, and 18 months (Figure 3A, *P < *.001). Thereafter we further classified the group of POCT and POCRT, the Median LRFS were, respectively, 33, 31, 24, 11 months in group POCT (Figure 3B, *P *< .001), and 39, 35, 28, and 27 months in group POCRT (Figure 3C, *P* = .072). In general, patients with multiple‐station pN2 were more prone to local recurrence than single‐station pN2. Moreover, patients with single‐station pN2 + multiple‐station pN1 had a relatively short LRFS with median LRFS was 24 months. Our study also showed that PORT remarkably increased the LRFS of the group multiple‐station pN2 ± pN1 (27 m versus 11 m), it also had a relatively longer LRFS in the single‐station pN2 + pN1 group, due to the limited sample of patients, LRFS was not significantly prolonged in the other groups. Details are described in Figure S1.

### 3.4 Multivariate analysis for LRFS of 183 patients

The clinicopathologic factors which may affect local recurrence for patients in group POCT have been described in Table 2. In univariate analysis, pathologic T stage, age, size of the primary tumor, tumor histological differentiation degree, the number of pN2 nodal stations, the number of positive pN2, the ratio of positive pN2 were significantly correlated with local recurrence. Multivariate analyses were performed to detect the association between the pN2 nodal stations and LRFS (multiple levels versus single level, HR, 1.793 [95% CI, 1.212‐2.652], *P* = .003), PORT (Yes or No, HR, 0.499 [95% CI, 0.363‐0.684], *P* < .001) and the ratio of positive pN2 has also been investigated and remained significant for LRFS (>1/3 versus ≤1/3 HR, 1.530 [95% CI, 1.045‐2.238], *P* = .029). In addition, Tumor histological differentiation degree (HR, 1.553 [95% CI, 1.184‐1.985], *P* = .001) and age (>55 y versus ≤55 y HR, 1.407[95% CI, 1.073‐1.911], *P* < .028) were significant predictors of LRFS.

**Table 2:** Univariate analysis and multivariate analysis about locoregional recurrence factors [n (%)]

Characteristic	Total (n = 183)	Univariate analysis		Multivariate analysis		
		*χ* b	*P* a	HR^3^	95%CI^4^	*P* b
Gender		0.542	.462		ND^5^	
Male	117 (63.9)					
Female	66 (36.1)					
Age		6.359	.012	1.407	1.073‐1.911	.028
Y ≤ 55 y	92 (50.3)					
Y> 55 y	91 (49.7)					
Histology		0.023	.989		ND	
Squamous Carcinoma	54 (29.5)					
Adenocarcinoma	111 (60.7)					
Others	18 (9.8)					
Smoke		0.186	.666		ND	
Yes	101 (55.2)					
No	82 (44.8)					
Operation		0.370	.543		ND	
Open operation	117 (63.9)					
Endoscopic surgery	66 (36.1)					
Adjuvant chemotherapy		6.016	.014	0.727	0.502‐1.052	.110
Cycle < 4	45 (24.6)					
Cycle ≥ 4	138 (75.4)					
Differentiation degree		6.705	.035	1.533	1.184‐1.985	.001
Well	19 (10.4)					
Moderately	104 (56.8)					
Poorly	60 (32.8)					
T stage		6.678	.035	1.108	0.767‐1.603	.343
T1	81 (44.3)					
T2	74 (40.4)					
T3	28 (15.3)					
T size		4.889	.027	1.056	0.634‐1.759	.380
T ≤ 3 cm	82 (44.8)					
T> 3 cm	101 (55.2)					
N2		17.777	<.001	1.793	1.212‐2.652	.003
Multiple station N2	103 (56.3)					
Single station N2	80 (43.7)					
Ratio of positive LN		24.673	<.001	1.530	1.045‐2.238	.029
Ratio ≤ 1/3	101 (55.2)					
Ratio > 1/3	82 (54.8)					
Number of positive LN		10.716	.001	1.080	0.693‐1.683	.790
Number ≤ 3	65 (35.6)					
Number > 3	118 (64.4)					
PORT		14.618	<.001	0.499	0.363‐0.684	<.001
Yes	78 (42.6)					
No	105 (57.4)					
LN7		1.203	.273		ND	
Yes	74 (40.4)					
No	109 (59.6)					
Vascular infiltration		3.384	.066		ND	
Yes	9 (4.9)					
No	174 (95.1)					

Abbreviations: CI, confidence interval; HR, hazard ratio; ND, No Done.

a Kaplan‐Meier.

b Cox.

### 3.5 Pattern of local recurrence in group POCT

During the course of the disease, 50 patients (47.6%) (50/105) in group POCT represented a first site or cumulative LR, 147 recurrent sites were observed in the POCT group (2.94 sites per patient). The highest risk site of failure was the ipsilateral hilum (21.0%), the bronchial stump (20.0%), followed by LNs4R (19.0%), LNs4L (18.1%), LNs7 (15.2%). In addition, Table 3 showed the distribution of LR in different sites of the initial tumor in detail, outcomes were as follows.

**Table 3:** The relationship between primary site and failure pattern in group POCT and POCRT [n (%)]

Lobe SLN	POCT							Total	POCRT							Total
	RUL	RML	LUL	LLL	RLL	LL	RL		RUL	RML	LUL	LLL	RLL	LL	RL	
N	33	13	21	17	21	38	67	105	26	4	15	17	16	32	46	78
1	1	1	3 (14.3)	1	2	4	4	8	1	0	1 (6.67)	3 (17.6)	1 (6.25)	4 (12.5)	2	6 (7.69)
2L	1	0	1	2	0	3	1	4	0	0	0	0	0	0	0	0
2R	6 (18.2)	1	1	2	2	3	9	12	1	0	0	1	0	1	1	2
3	1	0	0	1	1	1	2	3	0	0	0	1	0	1	0	1
4L	4	1	6 (28.6)	5 (29.4)	3 (14.3)	11 (28.9)	8	19 (18.1)	3 (11.5)	0	2 (13.3)	2 (11.8)	0	4 (12.5)	3 (6.50)	7 (8.97)
4R	7 (21.2)	2 (15.4)	3 (14.3)	3 (17.6)	5 (23.8)	6 (15.8)	14 (20.9)	20 (19.0)	3 (11.5)	0	1 (6.67)	1	0	2 (6.25)	3 (6.50)	5 (6.41)
5	2	0	5 (23.8)	3 (17.6)	3 (14.3)	8 (21.1)	5	13	2 (7.69)	0	1 (6.67)	0	0	1	2	3
6	2	0	3 (14.3)	3 (17.6)	0	6 (15.8)	2	8	1	0	1 (6.67)	1	0	2 (6.25)	1	3
7	4	2 (15.4)	3 (14.3)	3 (17.6)	4 (19.0)	6 (15.8)	10 (14.9)	16 (15.2)	1	0	0	0	0	0	1	1
8	0	0	0	1	0	1	0	1	0	0	0	0	0	0	0	0
Hilar	5 (15.2)	2 (15.4)	6 (28.6)	5 (29.4)	4 (19.0)	11 (28.9)	11 (16.4)	22 (21.0)	2 (7.69)	0	1 (6.67)	1	1 (6.25)	2 (6.25)	3 (6.50)	5 (6.41)
Stump	6 (18.2)	3 (23.1)	5 (23.8)	3 (17.6)	4 (19.0)	8 (21.1)	13 (19.4)	21 (20.0)	2 (7.69)	0	2 (13.3)	4 (23.5)	1 (6.25)	6 (18.8)	3 (6.50)	9 (11.5)

Abbreviations: LL, left lung; LLL, left lower lobe; LUL, left upper lobe; RL, right lung; RLL, right lower lobe; RML, right middle lobe; RUL, right upper lobe; SLN, Station of Lymph Nodes.

For the left upper lobe (n = 21), the most common failure site was LNs 4L and ipsilateral hilum (n = 6, 28.6%), followed by the bronchial stump, LNs 5, LNs 6, LNs7, LNs4R, and LNs1.

For the left lower lobe (n = 17), the most frequently involved recurrence sites were LNs 4L and the ipsilateral hilum (n = 5, 29.4%), followed by LNs 5, LNs 4R, the bronchial stump, LNs 7, and LNs6.

For the right upper lobe (n = 33), the highest failure site was 4R (n = 7, 21.2%), and it also had a high number in site of the bronchial stump, LNs 2R, and the ipsilateral hilum.

For the right middle lobe (n = 13), the most frequently involved recurrence site was the bronchial stump (n = 3, 23.1%), followed by the ipsilateral hilum, LNs 4R and LNs 7.

For the right lower lobe (n = 21), the most frequently involved site of failure was LNs 4R (n = 5, 23.8%), and it also had a high number in site of the ipsilateral hilum, LNs 7, the bronchial stump, LNs 5, and LNs 4L.

**Figure 4:**
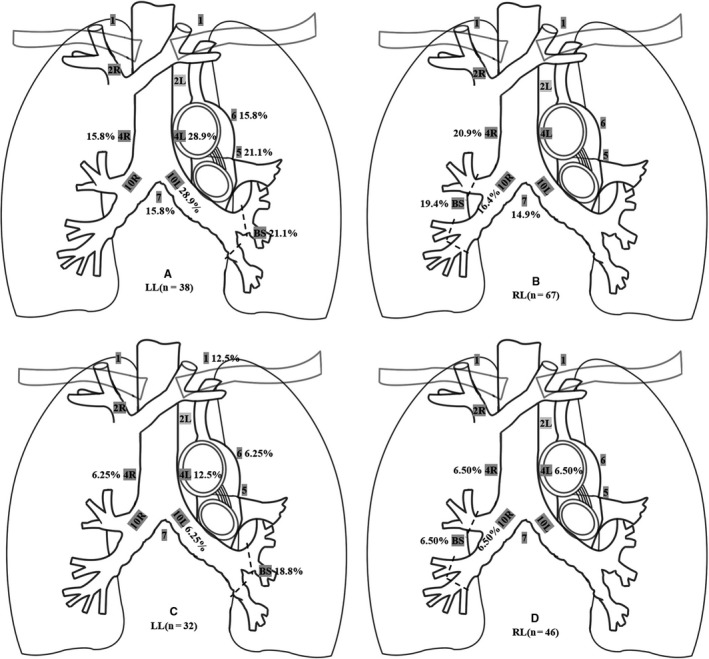
Locoregional recurrence pattern of left‐sided lung cancers (A) and right‐sided lung cancers (B) in group POCT; Locoregional recurrence pattern of left‐sided lung cancers (C) and right‐sided lung cancers (D) in group POCRT

For the left side lung (n = 38), the ipsilateral hilum (28.9%), LNs 4L (28.9%), the bronchial stump (21.1%), LNs 5 (21.1%), LNs 6 (15.8%), LNs 7 (15.8%), and LNs 4R (15.8%) were more prone to local recurrence, details described in Figure 4A.

For the right side lung (n = 67) the LNs 4R (20.9%), the bronchial stump (19.4%), the ipsilateral hilum (16.4%) and LNs 7 (14.9%) were more prone to local recurrence, details described in Figure 4B.

### 3.6 Pattern of local recurrence in group POCRT

POCRT offered a significantly lower risk for local recurrence when compared to group POCT. LR (first site or cumulative) was observed in 17 patients (21.8%) in the POCRT group. 42 nodal recurrent sites were discovered in the POCRT group (2.5 sites per patient). The most frequent failure site was the bronchial stump (11.5%), followed by LNs4L (8.97%), LNs1 (7.69%), the ipsilateral hilum (6.41%), and LNs4R (6.41%). Locoregional recurrence pattern of left‐sided lung cancers and right‐sided lung cancers were described in Figure 4C,D. It is worth noting that LR in LNs1 became frequent in the POCRT group and LNs1 was almost out of PTV, in addition, LR in LNs7 was decreased sharply in the POCRT group. LNs4L and LNs4R were prone to relapse regardless of the left and right‐sided tumors. Table 3 shows the distribution of LR in different sites of the initial tumor in detail.

## 4 DISCUSSION

Our retrospective study results demonstrate a significant improvement in the median LRFS and a trend toward longer survival in the POCRT versus POCT group. In addition, the ratio of LR in group POCRT was lower than group POCT and more sites of recurrent were found in group POCT compared with group POCRT (2.94 vs 2.5 sites per patient), suggesting indirectly benefits of PORT. The outcomes are not in accordance with the meta‐analysis^6^ in 1998, which indicated a 7% absolute increase in mortality associated with PORT, this is presumably due to the antiquated techniques, doses and large RT fields that increased the likelihood of cardiac and pulmonary toxicity. However, with the application of IMRT or 3DCRT‐based PORT, it might play an important role in the treatment of N2 disease. Of note, the SEER database^19^ analysis showed that PORT provides a noticeable survival benefit for patients with N2 nodal disease (*P* = .0077), similar findings were claimed in an ANITA trial^20^ subset analysis in 2008. Another retrospective study reported that a higher 5‐year OS rate (36.6% vs 30.6%; *P* < .046) with PORT was observed.^21^ A recent publication from Herskovic et al^7^ shows the conclusion that use of PORT was beneficial to the NCDB and was also noted in a prior study by Robinson et al^5^ Modern PORT seems to confer an additional OS advantage in the treatment of N2 disease. Nevertheless, based on the outcomes of META studies,^8^ PORT offered a significantly lower risk for local recurrence, but this effect does not translate into an obvious OS benefit. Therefore, the impact of PORT for resected pN2 NSCLC in the setting of standard adjuvant chemotherapy remains controversial. Lung Adjuvant Radiotherapy Trial^9^ (Lung ART), the ongoing randomized phase III trial, focus on evaluation the modern PORT in pN2 NSCLC, will hopefully shed new light on this dilemma. It is worth noting that a randomized, open‐label, phase III trial, ADJUVANT^22^ demonstrated patients with resected EGFR‐mutant, stage II–IIIA NSCLC, adjuvant gefitinib prolonged recurrence time and showed advantages over VP chemotherapy (28.7 versus 18.0 months; *P* = .0054). Adjuvant TKIs may be considered a treatment option in the resected stage N2 EGFR‐mutant NSCLC, a big challenge for PORT.

In fact, patients with pN2 NSCLC can be considered as a heterogeneous group with various clinicopathological features. Therefore, the current research is aiming to identify and validate the high risk patients who would be optimally suitable for postoperative radiation therapy. Previous studies^23^ have suggested that patients with skip lymph node metastases tend to have a better prognosis than other pN2 patients. The number of metastatic pN2 lymph nodes is an independent prognostic indicator in patients with curatively resected pN2 NSCLC.^10^, ^11^ Urban et al claimed that the LN ratio has been considered a practicable prognostic metric in NSCLC,^24^ and a similar outcome was seen in the previous article,^25^ indicating a high LN ratio that predicts the benefit of PORT. Moreover, the extent of resection, size of the primary tumor,^26^ the extracapsular spread in N2 disease^10^ and so forth, might be predictive for the prognosis of the NSCLC patients. Based on our analysis, patients with multiple pathologically N2 with a higher risk of local failure, for the group single‐station pN2 + multiple‐station pN1, it shows relatively shorter LRFS, we may treat these two groups more aggressively with PORT. According to the other findings of our study, patients with a high positive LN ratio ≥ 1/3 and a poor tumor histological differentiation degree might indicate a worse LRFS, PORT was more suitable for them. In short, subclassification according to the number or proportion of positive metastatic lymph nodes may be more appropriate in pN2 disease which has more heterogeneous prognostic features. Moreover, the pathologists and surgeons were in charge of the estimation of the number of metastatic lymph nodes, and subjective variations might be large, especially in cases of fragmented nodes or a conglomerate of matted nodes. This might be a limitation.

Although previous research has reported the rates of local recurrence in detail, patterns of failure are rarely described. It leads to a general unawareness of recurrence patterns, which may relate to the choice of adjuvant treatment modalities.^12^ Therefore, we attempt to investigate the locoregional patterns of relapse after induction chemotherapy followed by surgical resection in patients with pN2 NSCLC and try to explore the optimal PORT CTV in this cohort of patients. According to our research, most of the ipsilateral recurrences dominated for right‐sided tumors, whereas left‐sided tumors more frequently represented the contralateral mediastinum, this phenomenon occurs commonly in LNs4R. This may be determined by routes of lymphatic drainage of a different pulmonary lobe, for right‐sided tumors, these pathways constantly result in ipsilateral paratracheal, however, the metastatic pathway of left lung cancer is complex, prevascular, paraaortic, and the AP window is frequently involved.^27^ So far, several typical findings are worthy of our attention, Kelsey et al 27 have illustrated the location and distribution of local recurrent sites in different pulmonary lobe, which was basically consistent with our conclusion. The findings of Feng et al,^13^ the most common LNs failure site was 4R, followed by 7, 4L, 6, 10L, and 5 left‐sided lung cancer, For right‐sided lung cancer, the most common site of failure was station 2R, followed by 10R, 4R, and 7. In 2016, Billiet et al^28^ showed LRs were mostly seen in the LNs7 (18%), 4R (16%), and 10R (16%), and mainly bilaterally LR relapse pattern in left‐sided tumors, whereas in right‐sided tumors LN occurred more unilateral. Depending on the pattern of relapse, we may conclude that PORT CTV may vary depending on the lung lobe in which the tumor is located. In another study published in 2010,^16^ the article summarized in detail which lymph node regions needed to be included in the delineation of CTV for different positive lymph nodes after surgery, LNs4, LNs7 were basically included in every delineation of CTV. 2018 ESTRO ACROP^29^ guidelines suggested that the CTV consists of the resected involved anatomical mediastinal lymph node regions, the bronchial stump, the ipsilateral hilum, nodal stations 4 and 7. Hence, regardless of the conclusions of our study or the recommendations of the guidelines, the definition of CTV might depend on the lung lobe where the tumor is located, except the bronchial stump, the ipsilateral hilum and positive lymph node regions, LNs4, LNs7 may be considered within CTV due to their frequently relapsed. In addition, for the upper lobe, LNs2 was frequently involved and for the left lobe, the AP window also frequently relapsed and should be included in the radiation field.

In conclusion, indirect evidence shows that the efficacy of PORT in eradicating microscopic tumor cells after surgery. In our study, LRs occurred as the first site and concomitant with a distant relapse was (36.1%) (38/105) in group POCT and 16.7%（13/78）in group POCRT, most of the patients with an LR also had DMs (distant metastasis) the time of diagnosis of an LR, the ratio in group POCT and POCRT was respectively, 76.0% versus 76.5%. These patients tend to have larger primary lesions, more lymph node stations, vascular invasions and so on. Such patients may require a more effective local and systemic treatment. Due to the limited sample of patients, we did not continue to explore in this study. We look forward to more representative research in the future to find the commonality of such patients so as to find the most reasonable treatment for the patients with LR and DMs simultaneously.

There are limitations to our analysis. First, the retrospective nature of our study, there is no standardization in follow‐up intervals and radiological evaluation at the time of relapse. Furthermore, the quality of “complete resection” for lung cancer surgery may be heterogeneous due to vary surgeons, the reassessment of PORT should take the quality of surgery into consideration. In addition, the subjective variations might be large when the pathologists estimate the number of metastatic lymph nodes, and especially in cases of fragmented nodes or a conglomerate of matted nodes. Another limitation is almost all patients with recurrence were diagnosed by CT; pathological confirmation was not available for them. Finally, because of the limited patient number, it might be difficult for us to draw certain conclusions toward delineation of target volumes.

## 5 CONCLUSION

In conclusion, PORT leads to a strikingly reduced risk of local recurrence and a demonstrable OS benefit in our research. Patients with pN2 NSCLC have been considered as a heterogeneous group, for the group multiple‐station pN2, group single‐station pN2 + multiple‐station pN1, patients with a high positive LN ratio > 1/3 and a poor tumor histological differentiation degree might be more suitable for PORT and a more effective system therapy. The definition of CTV might be based on the lung lobe in which the tumor is located, except the bronchial stump, the ipsilateral hilum and positive lymph node regions, LNs4, LNs7, LNs5, and LNs6 may be considered within the CTV for the left‐side lung, LNs4R and LNs7 should be considered within CTV for the right‐side lung.

## CONFLICTS OF INTEREST

The author reports no conflicts of interest in this work.

## AUTHOR CONTRIBUTIONS

Conceptualization, ZGL, Data curation, W.W and J.Z; Formal analysis, Q.Z and DHL; Investigation, QDL; Methodology, BLZ and ZBL; Resources, YCZ; Software, RJ and GYL; Supervision, CYX and SYZ; Validation, HLZ and J. W; Visualization, N.Y; Writing – review and editing, W.W and J. Z.

## Data Availability

The data that support the findings of this study are available from the corresponding author upon reasonable request.
