# Supplementary material for: Postoperative intensity‐modulated radiation therapy reduces local recurrence and improves overall survival in III‐N2 non‐small‐cell lung cancer: A single‐center, retrospective study
Source: Cancer Med. 2020 Feb 26;9(8):2820–32. doi: 10.1002/cam4.2937 (PMC7163098; doi:10.1002/cam4.2937)
Supplement: Supplementary file 1 [file CAM4-9-2820-s001.pptx]

## Slide 1
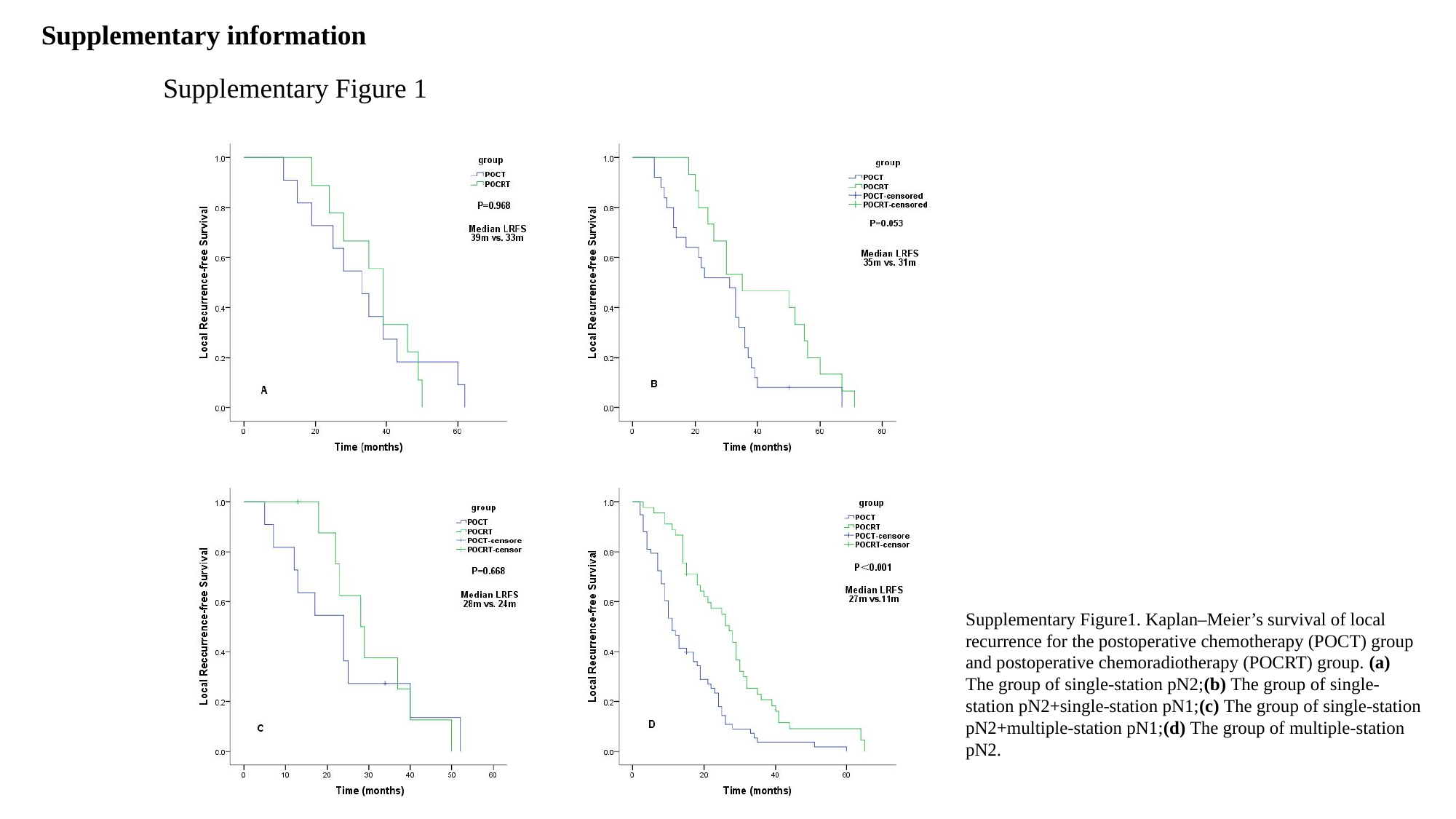

Supplementary information
Supplementary Figure 1
Supplementary Figure1. Kaplan–Meier’s survival of local recurrence for the postoperative chemotherapy (POCT) group and postoperative chemoradiotherapy (POCRT) group. (a) The group of single-station pN2;(b) The group of single-station pN2+single-station pN1;(c) The group of single-station pN2+multiple-station pN1;(d) The group of multiple-station pN2.
